# Developing Novel Fabrication and Optimisation Strategies on Aggregation-Induced Emission Nanoprobe/Polyvinyl Alcohol Hydrogels for Bio-Applications

**DOI:** 10.3390/molecules27031002

**Published:** 2022-02-02

**Authors:** Javad Tavakoli, Jesus Shrestha, Sajad R. Bazaz, Maryam A. Rad, Majid E. Warkiani, Colin L. Raston, Joanne L. Tipper, Youhong Tang

**Affiliations:** 1 Centre for Health Technologies, School of Biomedical Engineering, Faculty of Engineering and Information Technology, University of Technology Sydney, Sydney, NSW 2007, Australia; javad.tavakoli@uts.edu.au (J.T.); jesus.shrestha@student.uts.edu.au (J.S.); sajad.razavibazaz@student.uts.edu.au (S.R.B.); maryamalsadat.rad@student.uts.edu.au (M.A.R.); majid.warkiani@uts.edu.au (M.E.W.); 2 Institute for NanoScale Science and Technology, College of Science and Engineering, Flinders University, Adelaide, SA 5042, Australia; colin.raston@flinders.edu.au; 3 Medical Device Research Institute, College of Science and Engineering, Flinders University, Adelaide, SA 5042, Australia

**Keywords:** fluorescent nanoprobe, hyperbranched polymers, aggregation-induced emission, vortex fluidic device, fluorescent hydrogel

## Abstract

The current study describes a new technology, effective for readily preparing a fluorescent (FL) nanoprobe-based on hyperbranched polymer (HB) and aggregation-induced emission (AIE) fluorogen with high brightness to ultimately develop FL hydrogels. We prepared the AIE nanoprobe using a microfluidic platform to mix hyperbranched polymers (HB, generations 2, 3, and 4) with AIE (TPE-2BA) under shear stress and different rotation speeds (0–5 K RPM) and explored the FL properties of the AIE nanoprobe. Our results reveal that the use of HB generation 4 exhibits 30-times higher FL intensity compared to the AIE alone and is significantly brighter and more stable compared to those that are prepared using HB generations 3 and 2. In contrast to traditional methods, which are expensive and time-consuming and involve polymerization and post-functionalization to develop FL hyperbranched molecules, our proposed method offers a one-step method to prepare an AIE-HB nanoprobe with excellent FL characteristics. We employed the nanoprobe to fabricate fluorescent injectable bioadhesive gel and a hydrogel microchip based on polyvinyl alcohol (PVA). The addition of borax (50 mM) to the PVA + AIE nanoprobe results in the development of an injectable bioadhesive fluorescent gel with the ability to control AIEgen release for 300 min. When borax concentration increases two times (100 mM), the adhesion stress is more than two times bigger (7.1 mN/mm^2^) compared to that of gel alone (3.4 mN/mm^2^). Excellent dimensional stability and cell viability of the fluorescent microchip, along with its enhanced mechanical properties, proposes its potential applications in mechanobiology and understanding the impact of microstructure in cell studies.

## 1. Introduction

Hydrogels are three-dimensional cross-linked networks with the ability to uptake large amounts of water, i.e., up to 1000 times, of their dry weight [1]. Depending on the cross-linking methods, i.e., physical or chemical, these hydrophilic polymers may be chemically stable or degrade over time, such that each can serve for a specific application [2,3,4]. From simple structures to smart networks, various types of enviro-sensitive hydrogels have been introduced over the years. Specifically, thermo-responsive and pH-sensitive hydrogels have been employed for targeted delivery and sustained release of biological compounds [5,6,7]. These hydrogels generally require different environmental stimulators, such as magnetic and electric fields [8,9], biomolecule concentration [10,11], and temperature [12,13] to control their swelling properties. Meanwhile, extensive efforts have been put to prepare unique hydrogels with improved physical, mechanical, and biomedical properties for a wide range of applications [14,15,16].

Recent initiatives to develop a new class of hydrogels have been mostly focused on producing fluorescent hydrogels [17,18,19]. The ability of the fluorescent hydrogel to emit light under a specific excitation wavelength makes them attractive for different applications, including sensors, bioimaging, and fabrication of tissue-engineered scaffolds with specific features [20,21]. Swelling a hydrogel in a dye solution and physical mixing of polymer with a fluorophore have been frequently used for the preparation of fluorescent hydrogels. However, most fluorophores are bulky and hydrophobic; therefore, their physical introduction to hydrogel structures can affect the chemical and mechanical stability of hydrogels. Moreover, the aggregation of fluorophores results in fluorescence quenching, known as aggregation, caused quenching, and reduces the fluorescence intensity leading to negligible brightness [22,23,24]. Overall, the generation of bright and stable fluorescent hydrogels for practical applications remains an arduous challenge.

The recent discovery of aggregation-induced emission fluorogens (AIEgens) with high emission efficacy in the aggregated state, unique photostability, noticeable diversity of molecular structures, and acceptable sensitivity have demonstrated new opportunities to overcome the major drawback [25]. AIEgens have been widely used for the preparation of fluorescent hydrogels [26,27]. Indeed, AIEgens exhibit enhanced fluorescent properties within a confined polymer network due to the restriction of their intramolecular rotation occurring via chemical or physical interactions. In addition, few studies have employed AIEgens for hydrogel characterization, such as visualization of crystalline regions [28], accurate measurement of the swelling [29], and monitoring the degradation processes [30] features that were not attainable before. While AIEgens have provided excellent opportunities to create hydrogels with superior fluorescent properties, the major challenge here is that synthesis of such polymer gels is currently problematic, involving multi-step processes and complex chemistry, limiting large-scale production [31].

The current study presented an innovative strategy to fabricate fluorescent hydrogels using a vortex fluidic device (VFD) to address the associated challenges. VFD is a new microfluidic platform that comprises a fast rotating tube to create a dynamic thin film imparting high shear force in the form of submicron topological fluid flow, and micro-mixing [32]. Typically, a glass tube that is closed at one end (45° tilt angle) with a finite amount of material is rapidly rotated, as the confined mode of the VFD, or where materials are constantly fed into the tube, exiting at the top, as the continuous flow mode of operation. VFD processing, compared to simple mixing (i.e., magnet stirrer), proposed a range of benefits leading to improved control over chemical reactions and material synthesis [33]. The VFD has been used for a number of diverse applications including the fabrication of various nanocarbon materials, protein folding and purification, polymer networks manipulation, and graphite exfoliation [34,35,36]. The microfluidic platform is capable of controlling the size and shape of nanoparticles for both top–down and bottom–up processing [37,38,39]. Our previous studies revealed that VFD tuned the size of AIEgens [40] and introduced a new and cost-effective approach for the fabrication of AIE nanoprobes based on the formation of strong hydrogen bonds between the hyperbranched polymer (HB) and AIEgen (AIE) [41]. These studies identified the effect of AIEgen concentration and water fraction on the fluorescent properties of the AIE-HB; however, the impact of the type of hyperbranched polymers on the associated fluorescent properties is yet to explore. Motivated by the above research, the aims of the current study were two-fold. The first aim was to identify the effect of the type of HB on the fluorescent properties of the AIE-HB nanoprobe to further understand the impact of hyperbranched molecular structure. The second aim of this study was to employ the nanoprobe with optimum fluorescent properties to create fluorescent hydrogels and evaluate their physicochemical, mechanical, and biomedical properties. For the second aim, two different case studies were considered. The first case study was developing an injectable bioadhesive gel based on polyvinyl alcohol (PVA) crosslinked by borax, exhibiting fluorescent properties. For the second case study, a microfluidic fluorescent device (microchip) containing complex microstructure was prepared relying on physical crosslinking of PVA [42,43,44,45]. The current fluorescent microfluidic device can be used as a cell culture platform allowing the evaluation of the impact of surface morphology on cell properties.

While these case studies involved PVA hydrogel, our innovative technique may be potentially used for the fabrication of a series of fluorescent hydrogels, with their creation relying on the formation of hydrogen bonds including but not limited to PVA [46,47], gelatin, starch [48,49], cellulose [50,51], guar gum [52,53], and their composites. To the best of our knowledge, the current study is the first research of its kind aimed to introduce a technique for the fabrication of fluorescent hydrogels using a VFD.

## 2. Results and Discussion

### 2.1. Nanoprobes Characterization

#### 2.1.1. The Generation of HBs Affected the Fluorescent Property of the AIEgen

**Figure 1:**
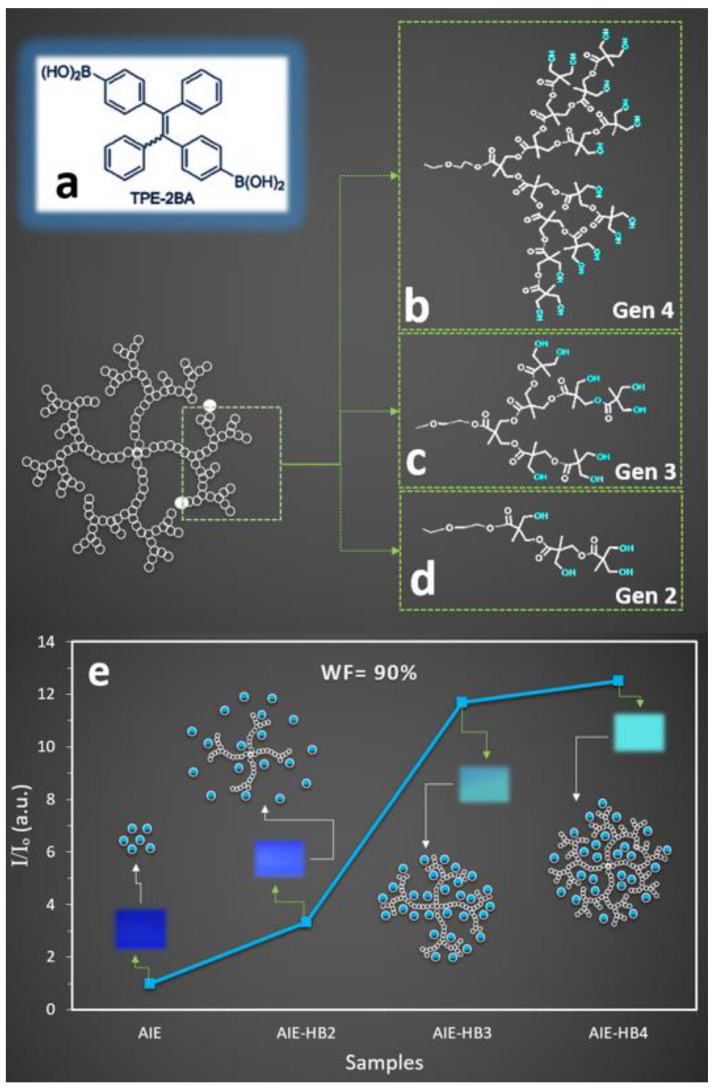
Chemical structures of (**a**) TPE-2BA (AIE) and schematic drawing of different types of the hyperbranched polymers (HBs) including the chemical structure of one branch only for, (**b**) generation 4, (**c**) generation 3, and (**d**) generation 2. (**e**) Change in relative fluorescence intensity of TPE-2BA-hyperbranched polymer (AIE-HB nanoprobe) at water fraction = 90% with AIE (blue circles) and HB (white circles) representing AIEgen and HB, respectively. The concentrations of TPE-2BA and all different generations of HBs were 50 μM and 1 mM, respectively, and the excitation wavelength was 310 nm. Insets: camera images of AIE and nanoprobe solutions at WF = 90% captured under a UV light with the excitation wavelength of 365 nm.

Our previous study revealed that the fluorescence intensity of conventionally prepared TPE-2BA (Figure 1a) particles was maximum for water fraction (WF) = 90%, then decreasing towards lower WFs with negligible fluorescence intensity for WF < 70% [28]. Therefore, the current study investigated the impact of three different types of HBs (Figure 1b–d) in the fluorescent property of the AIE at WF = 90%. When AIE-HBs were prepared under constant stirring (conventional process), an increase in the fluorescence property was observed for all samples (Figure 1e). Significant differences in the fluorescence intensity were observed for samples containing different generations of HB (concentration = 1 mM) at constant WF = 90%. The fluorescence intensity increased 13-fold when HB generation 4 was used compared to the AIE alone. We also found that the associated maximum fluorescence intensity increased approximately 3-fold and 12-fold, respectively, for HB generations 2 and 3 compared to the AIE alone. The incorporation of different generations of the HB led to an increase in the relative intensity, by approximately four-times for generation 3 compared to generation 2. Moreover, a negligible change in the fluorescence intensity (7% enhancement) was observed for nanoprobe generation 4 compared to that of generation 3.

#### 2.1.2. VFD Enhanced the Fluorescent Property of the AIE-HB Nanoprobes

**Figure 2:**
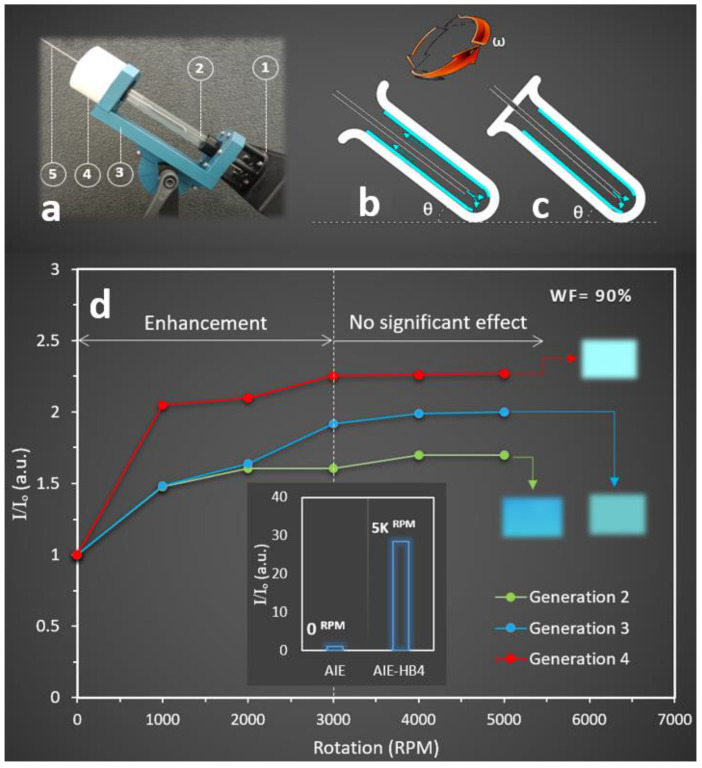
(**a**) Camera image of a VFD indicating its compartments: 1—electrical motor, 2—quartz tube, 3—base, 4—sample collector, and 5—jet feed inlet. Schematic drawing of a VFD tube representing (**b**) continuous flow and (**c**) confined modes with θ and ω representing the tilt angle and rotation speed of the tube, respectively. (**d**) The effect of rotation speed on the relative fluorescence intensity of nanoprobes (generations 2, 3, and 4) at the excitation wavelength of 310 nm. Insets: camera images of nanoprobe solutions at WF = 90% captured under a UV light with the excitation wavelength of 365 nm, and relative fluorescence intensity for nanoprobe generation 4 prepared using a VFD @5000 RPM compared to the AIE alone.

VFD is a microfluidic platform generating a dynamic thin film through a fast rotating tube ranging from 0 to 10,000 RPM to precisely control the film thickness, imparting high shear stress and micro-mixing (Figure 2a) [54]. Involving mechanical energy to control chemical reactions in a VFD can be controlled in confined and continuous flow modes of operations. In a continuous flow mode, chemicals are continuously fed into the rotating tube at predetermined flow rates, and the product collecting at the top (Figure 2b), while in a confined mode, a limited amount of chemicals are injected into the closed tube at once, permitting sample collection at the end of the process (Figure 2c). The VFD is a relatively low-cost research platform allowing material synthesis [55], tuning the size and structure of complex systems [56], and regulating chemical reactions [35] with a variety of advantages over conventional technologies [57]. In the current study, a VFD with a confined mode of operation was employed, resulting in the production of brighter and more stable AIE-HB than conventional processing.

When a VFD was utilised and the mixture of AIE (concentration = 50 µM) and the HB solutions (concentration = 1 mM) was added to water (WF = 90%) in a rotating VFD tube (1000–5000 RPM), an increase in the fluorescent property of the nanoprobe solution was observed. The effects of different rotation speeds on the fluorescence intensity of the nanoprobes were also examined. We found that nanoprobes prepared under shear stress in the VFD tube exhibited significantly higher fluorescence intensity than that of AIE particles. We observed that increasing the rotation speeds from 1000 to 3000 RPM increased the relative fluorescence intensity for all HB generations. However, the relative fluorescence intensity remained approximately unchanged for rotation speeds >3000 RPM (Figure 2d). We also found that the highest fluorescence intensity occurred at the rotation speed of 3000 RPM for the HB generation 4, with 50% and 12.5% enhancement compared to that of the HB generations 2 and 3, respectively.

**Figure 3:**
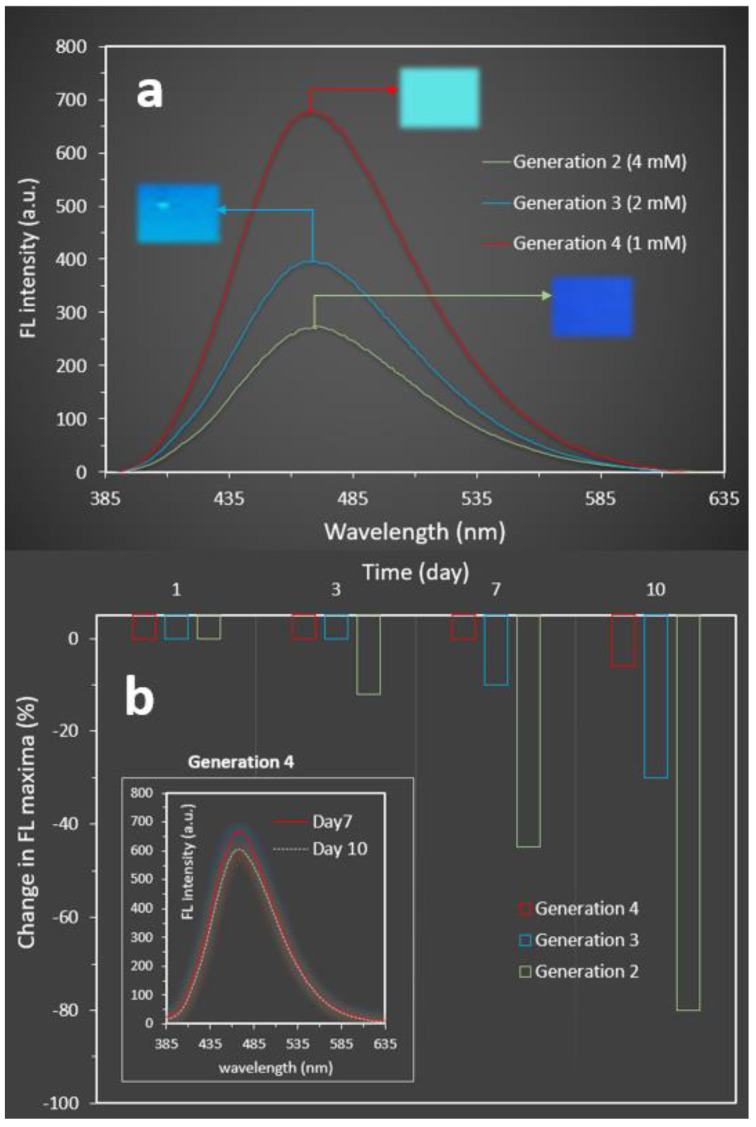
(**a**) Changes in the fluorescence intensities versus the concentrations of HBs to obtain an equal total number of OH groups (WF = 90%); insets: camera images of nanoprobe solutions captured under a UV light with the excitation wavelength of 365 nm). (**b**) The stabilities of the nanoprobes prepared using different generations of HB were measured over 10 days (at WF = 90%, concentration = 1 mM, and the excitation wavelength = 310 nm).

To further investigate the first aim, HB solutions with different concentrations of 1, 2, and 4 mM for generations 4, 3, and 2 were prepared, respectively. This approach enabled us to prepare HB solutions with an equal total number of hydroxyl groups (64 OH groups) per one AIE molecule. It was observed that increasing the concentration to reach an equal number of OH groups among all samples had no impact on the final fluorescence property. We observed that the peak fluorescence intensities for the HB generations 2 and 3 with concentrations of 4 and 2 mM, respectively, were lower than that observed for the HB generation 4 with a concentration of 1 mM (Figure 3a). This indicated the key role of the HBs’ chemical structure to restrict the AIE intermolecular rotation and tune the associated fluorescent properties. The results of the stability measurement identified that a more stable nanoprobe was achieved when HB generation 4 was employed (Figure 3b). Although the final brightness for the prepared nanoprobes using HB generations 3 and 4 was approximately similar (Figure 2d), the results for fluorescence stability revealed a less stable assembly for generation 3 with less than 3 days stability. When HB generation 2 was employed, the nanoprobe was not stable for more than 5 h.

**Figure 4:**
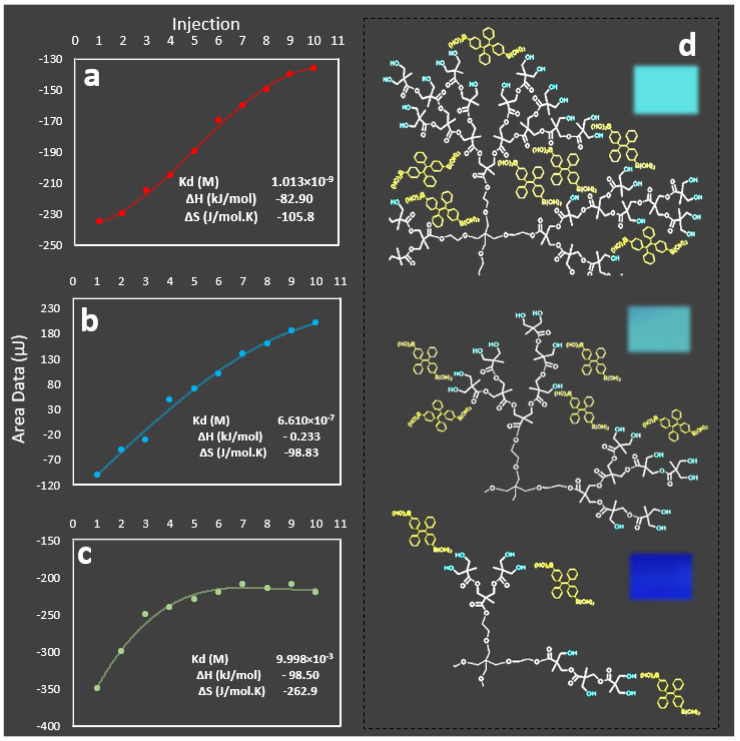
Isothermal titration calorimetry results for nanoprobes including (**a**) generation 4, (**b**) generation 3, and (**c**) generation 2, with estimated equilibrium dissociation constants using fitted model curves. (**d**) Schematic drawings for the interaction of AIE with different HB generations (only a part of each HB molecule was shown). Insets: camera images of nanoprobe solutions captured under a UV light with the excitation wavelength of 365 nm.

This observation was consistent with our previous studies, indicating that the penetration of AIE molecules into the HB structure to form hydrogen bonds, played a crucial role in both the formation and the stability of the nanoprobe. In further investigation, we measured the equilibrium dissociation constants when AIE was added to HB generations 2, 3, and 4 (Figure 4). It was observed that the equilibrium dissociation constant was higher for generation 4 (1 × 10^−9^) than those measured for generations 3 (6.6 × 10^−7^) and 2 (9.9 × 10^−3^).

### 2.2. Fluorescent Nanoprobe-Based Polymeric Hydrogel Fabrication and Characterization

The second aim of the current study was to develop a cost-effective technique to produce PVA-based fluorescent hydrogels using the prepared nanoprobe. The findings of this study revealed that developing an AIE-based nanoprobe using HB generation 4 exhibited maximum fluorescent property and optimum stability compared to HB generations 3 and 2. Two different case studies were considered to accomplish this aim. For the first case study, a fluorescent injectable gel was developed using borax solution as a crosslinking agent. A fluorescent PVA microchip was prepared based on physical cross-linking for the second case study.

#### 2.2.1. Case Study 1: Injectable Bioadhesive Fluorescent Gel

**Figure 5:**
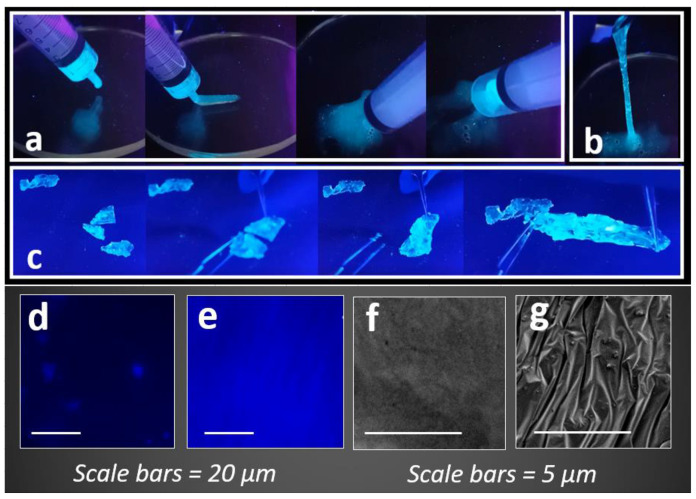
(**a**) The syringeability and (**b**) extensibility of the fluorescent PVA gel. (**c**) The self-healing property of the fluorescent PVA gel crosslinked with 100 mM borax solution. (**d**,**e**) Fluorescent microscope and (**f**,**g**) SEM images were captured from the surface of PVA + AIE and PVA + nanoprobe, respectively.

Our previous study identified that regulating the concentration of borax (50 mM) in a VFD tube with rotation speed = 4000 RPM resulted in creating PVA gels with self-healing properties [58]. Using similar properties, borax concentrations = 50 mM and VFD rotation speed = 4000 RPM, the current study investigated the role of the nanoprobe on the self-adhesion and fluorescent properties of the final gels, compared to PVA alone and traditionally prepared hydrogels (without VFD). It was observed that using a VFD, a highly extensible self-healed fluorescent gel was formed. The final fluorescent injectable gel exhibited acceptable syringeability with preserving its property under frequent filling and injection (Figure 5a,b). In contrast, it was not possible to fill a syringe with PVA + nanoprobe gel that was prepared using traditional processing (mixing without VDF using a magnet stirrer @ 1000 RPM), indicating the role of VFD in the construction of injectable and highly fluorescent PVA gel. Understanding the mechanism for the formation of the VFD-driven PVA gel, cross-linked with borax, was fully described in our previous study [58] and revealed that VFD imparts micromixing and shear forces at the sub-micron level to produce a more homogeneous gel. The results of the current study revealed that the addition of the nanoprobe to induce fluorescent property did not affect the syringeability of the final fluorescent gel when a VFD was used.

When the concentration of borax was increased to 100 mM and injectable gels were dried for 1 hr at room temperature, a fluorescent bioadhesive PVA gel was produced that exhibited self-healing properties (Figure 5c). To qualitatively evaluate the associated self-healing property, the fluorescent bioadhesive PVA gel was cut into pieces using a surgical blade. The pieces were then reattached using tweezers to avoid the application of large forces during reattachment. It was observed that the self-healed PVA gel appropriately exhibited a highly extensible feature after reattachment (Figure 5c).

The fluorescent microscopic images (Figure 5d,e) captured from the surface of injectable fluorescent gels revealed a higher brightness with almost uniform colour distribution for the PVA + nanoprobe gel compared to the PVA + AIE. This observation can be described by stronger hydrogen bonds that were formed between the AIEgen and the HB in the nanoprobe compared to the AIEgen alone. Consequently, a higher intermolecular rotation restriction was achieved for the nanoprobe that led to a significantly higher fluorescent property. This observation was evidenced by the lower equilibrium dissociation constant for the nanoprobe (K_d_ = 1 × 10^−9^) compared to the AIE alone. Our previous study identified that the dissociation constant for the AIE alone was K_d_ = 1 × 10^−5^ that was measured by injecting 25 µL of the AIEgen (50 µM) into DMSO [41]. Moreover, different surface morphologies were observed for the PVA + nanoprobe compared to the PVA + AIE bioadhesive gel (Figure 5f,g). This indicated that the introduction of the nanoprobe to the PVA structure induced more opportunities to form hydrogen bonds, leading to the development of a PVA bioadhesive gel with a layered structure imparting superior self-adhesion properties.

**Figure 6:**
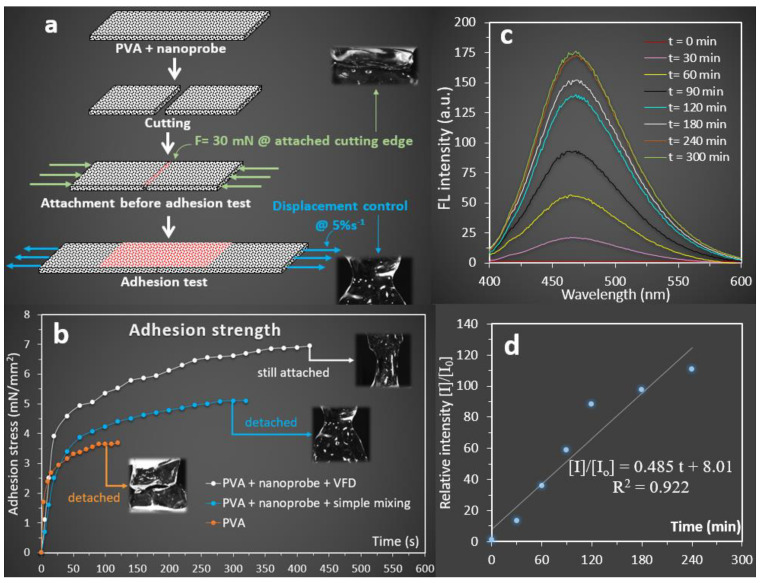
(**a**) Schematic drawings including camera images to describe the adhesion test procedure. (**b**) Comparison of the adhesion strength of PVA-based gels including PVA (blue line), traditionally prepared PVA + nanoprobe (blue line), and VFD-driven PVA + nanoprobe prepared at 4000 RPM (red line). Inset: camera images (×4) were captured during the measurement of the self-adhesion property. (**c**) Change in the fluorescent intensity of the release media showing the release of nanoprobe from the injectable bioadhesive PVA gel over time, and (**d**) change in relative fluorescent intensities of the release media at different time points, compared to t = 0 min, indicating almost a linear release behaviour for the gel.

The creation of highly extensible fluorescent PVA bioadhesive gel with excellent extensibility motivated us to quantify the associated adhesion properties. This approach enabled us to compare the adhesion property of the VFD-driven PVA + nanoprobe bioadhesive gel with PVA alone and the traditionally prepared PVA + nanoprobe (without VFD). All three samples were prepared using 100 mM borax and dried at room temperature for 1 h. The self-adhesion properties were measured at the cutting edge of the samples, identifying the adhesion stress during the detachment as schematically presented (Figure 6a). It was revealed that the detachment occurred at a 45% strain for the PVA bioadhesive gel, which was much lower than that measured for the traditionally prepared PVA + nanoprobe sample that exhibited 300% strain when detached. Detachment time was 120 s and 320 s for PVA and PVA + nanoprobe bioadhesive gel, respectively. After extension for 420 s, with a strain rate = 5% s^−1^, no detachment was observed at the cutting edges of the VFD-driven PVA + nanoprobe bioadhesive gel with the adhesion stress reaching 7.1 mN/mm^2^. The adhesion stresses for the traditionally prepared PVA + nanoprobe and PVA bioadhesive gel were approximately 5.1 and 3.7 mN/mm^2^ at the breaking point, respectively (Figure 6b). The results showed a 40% improvement for the adhesion stress when VFD was employed, compared to the traditional approach. Moreover, it was found that the incorporation of hyperbranched polymer and VFD in the preparation of PVA + nanoprobe gel led to an 87% improvement in adhesion stress (Figure 6b). Both injectable fluorescent (crosslinked with 50 mM borax) and bioadhesive (crosslinked with 100 mM borax) gels were not stable during swelling, and they disappeared during the process. Therefore, it was not possible to measure the swelling and mechanical properties and associated cell viability. It is more likely that further chemical cross-linking (i.e., glutaraldehyde) strengthens the hydrogel network; however, this may affect the associated biocompatibility.

We further measured the release of the nanoprobe from the injectable bioadhesive gel (Figure 6c,d) to understand the release behaviour of the nanoprobe. Our results revealed almost a linear trend (R^2^ = 0.922) for the release of nanoprobe for 300 min. This finding suggested that the injectable bioadhesive gel can potentially be used as a carrier for AIEgen delivery. The addition of AIEgens to enhance the fluorescent property of the self-healing gel will allow qualitative detection of release kinetics after injection, can tune the self-assembly property, and facilitate accurate gel injection under UV light exposure [59,60,61,62]. Of particular interest, TPE-2BA, the AIEgen that was used in this study, has been used to differentiate dead/live bacteria and detect glucose [63,64]. Therefore, the current fluorescent injectable bioadhesive gel is likely to have potential applications for developing an injectable glucose biosensor (transcutaneous) or smart wound dressing with the ability to detect bacterial infection.

#### 2.2.2. Case Study 2: Fluorescent Microchip for Cell Culture

**Figure 7:**
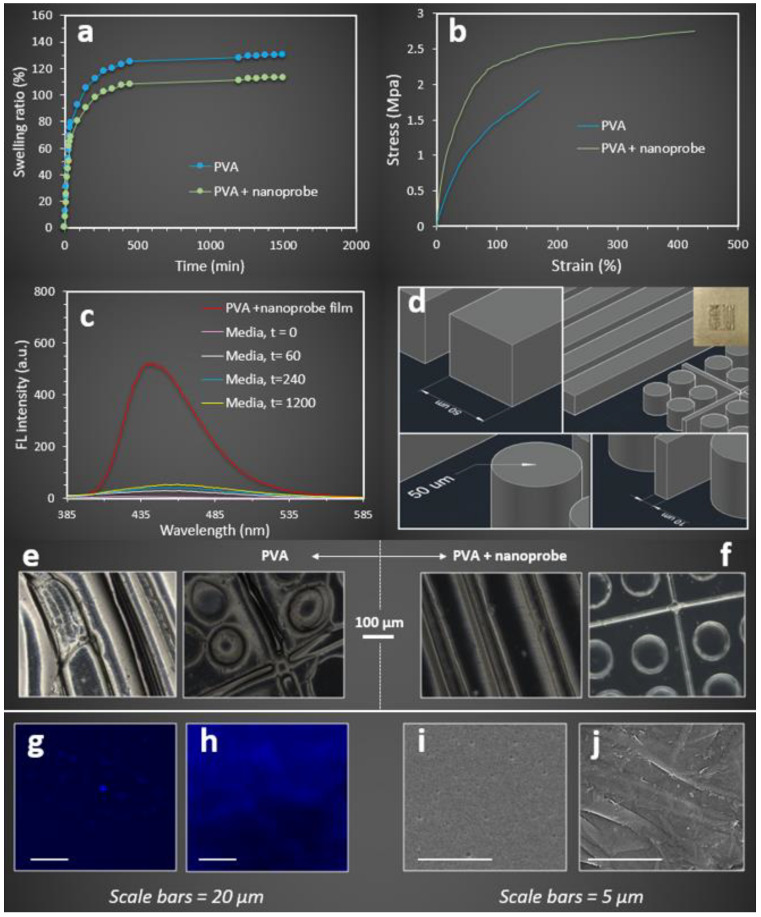
(**a**) Swelling and (**b**) mechanical properties of PVA and PVA + nanoprobe microchip. (**c**) Fluorescent spectra were collected from the PVA + nanoprobe microchip and swelling media (at different time steps) to quantify nanoprobe leakage during swelling. (**d**) SolidWorks drawings of complex 3D features to develop a platform containing different patterns using 3D printing technology. (**e, f**) Microscopic images of the surface of (**e**) PVA and (**f**) PVA + nanoprobe films indicate their structural stability during swelling in water. (**g**–**j**) Fluorescent and SEM images of the surface of PVA and PVA + nanoprobe films.

The swelling ratio of the fluorescent PVA microchip was determined by measuring the weight increase of samples after immersion in the distilled water over 24 h. The results of the swelling ratio revealed that the PVA-based microchip absorbed 125% water compared to its dry weight at the maximum swelling state after 450 min (Figure 7a). However, the addition of nanoprobe to the PVA structure led to a lower maximum swelling ratio (108%) compared to the PVA microchip. It was also observed that the initial water uptake rate and the time associated with the maximum swelling ratio remained approximately unchanged for both PAV and PVA + nanoprobe microchips. These observations were consistent with the mechanical properties measured for both microchips (Figure 7b). Higher mechanical properties were found for the PVA + nanoprobe compared to the PVA microchip. The PVA + nanoprobe microchip experienced 2.75 MPa maximum stress before failure, which was 44% higher than the PVA sample (1.84 MPa). The failure strains were 178% and 429% for the PVA and PVA + nanoprobe microchips, respectively. Young’s moduli for the PVA and PVA + nanoprobe microchips were 0.021 and 0.047 MPa, respectively. Moreover, a higher toughness was found for the PVA + nanoprobe (9.95 kJ·mm^−3^) compared to the PVA microchip (1.89 kJ·mm^−3^). Our mechanical characterization results revealed that the addition of the nanoprobe to the PVA structure significantly enhanced the mechanical properties of the microchip; hence, decreasing the swelling properties.

Our effort to further quantify the nanoprobe leakage from the PVA + nanoprobe microchip revealed that approximately 10% of the nanoprobes were expelled from the microchip after 20 h of swelling (Figure 7c). The maximum relative fluorescent intensity for the PVA + nanoprobe microchip was 578.4 (a.u.). The maximum relative fluorescent intensities for the swelling media were 0, 43.46, 56.14, and 59.31 after 0, 1, 4, and 20 h of swelling in water. This finding indicated that the leakage of the nanoprobe from the sample was minimal after 20 h of swelling. In addition, the PVA + nanoprobe microchip showed excellent dimensional stability during swelling. When PVA and PVA + nanoprobe microchips with a similar complex surface morphology (Figure 7d–f), were swelled (in water) to their maximum swelling ratio, a uniform volume expansion with negligible structural deformity was observed for the PVA + nanoprobe microchip (Figure 7f). In contrast, the dimensional stability of the PVA microchip was relatively weak, and structural deformities were found for all three different features (Figure 7e).

**Figure 8:**
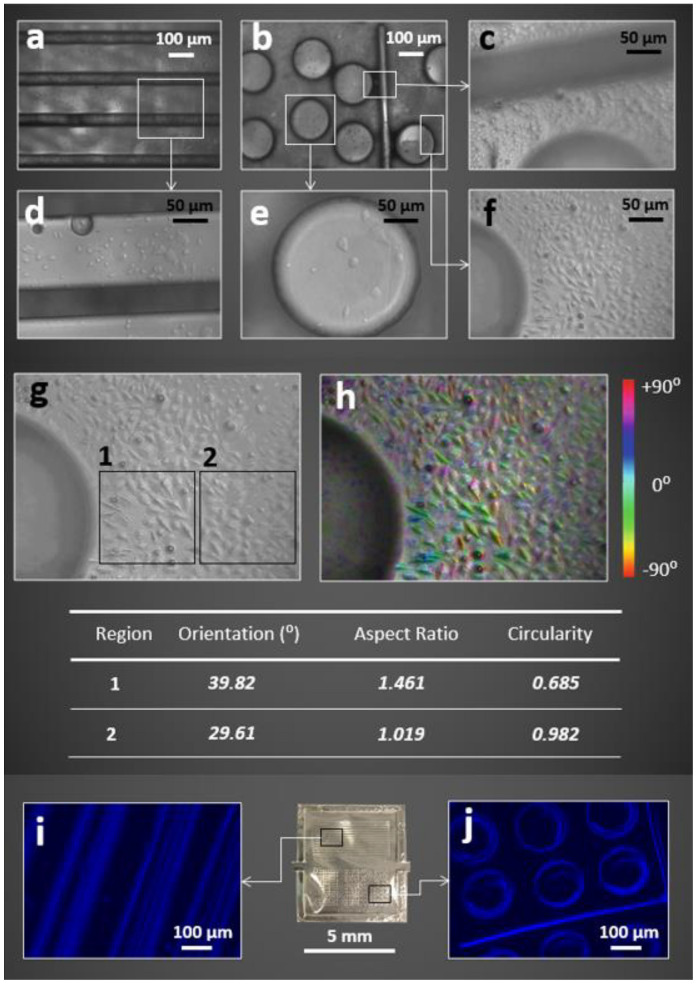
(**a**–**f**) Microscope images with different magnifications that were captured for different regions of the PVA + nanoprobe microchip in the cell culture media + L929 mouse cell media at their maximum swelling ratio. (**g**) A microscope image and (**h**) the corresponding image post-processing (ImageJ) to identify the impact of features on the orientation and shape of the cells. (**i**,**j**) Fluorescent microscope images of the microchip were captured from two different regions (denoted by black rectangles) of the microchip (middle camera image). In addition, we observed that the PAV + nanoprobe microchip provided a suitable microenvironment for cell culture studies. It was found that cell directionality was tuned by the 3D microstructure (**h**), both cell orientation and aspect ratio were smaller for the regions close to the microstructure. Microscope images that were captured from the surface of the PVA + nanoprobe microchip using a fluorescent microscope (350 nm) showed the fluorescent property of the swelled microchip (**i**,**j**).

The fluorescent microscopic images (Figure 7g,h) captured from the surface of fluorescent hydrogel films revealed a higher brightness with almost uniform colour distribution for the PVA + nanoprobe compared to the PVA film that was prepared by AIEgen alone. The capability of the nanoprobe to perform hydrogen bonds with PVA molecules to further restrict the intermolecular rotation seemed to play a key role in this finding. Different surface morphologies were observed for the PVA + nanoprobe compared to the PVA + AIEgen films (Figure 7i,j), indicating the impact of nanoprobe on the structural organization of PVA at the micro-level. The introduction of the nanoprobe to the PVA structure induced more opportunities for the formation of hydrogen bonds and created physical entanglements leading to the development of stiffer nanocomposite hydrogels with aligned surface morphology imparting superior mechanical and fluorescent properties. We further examined the dimensional stability of the PVA + nanoprobe microchip with a 3D complex surface morphology in cell culture media including L929 mouse cells, to investigate whether their stabilities differ compared to swelling in water (Figure 8). Microscopic images identified that PVA + nanoprobe microchips were dimensionally stable in cell culture media.

**Figure 9:**
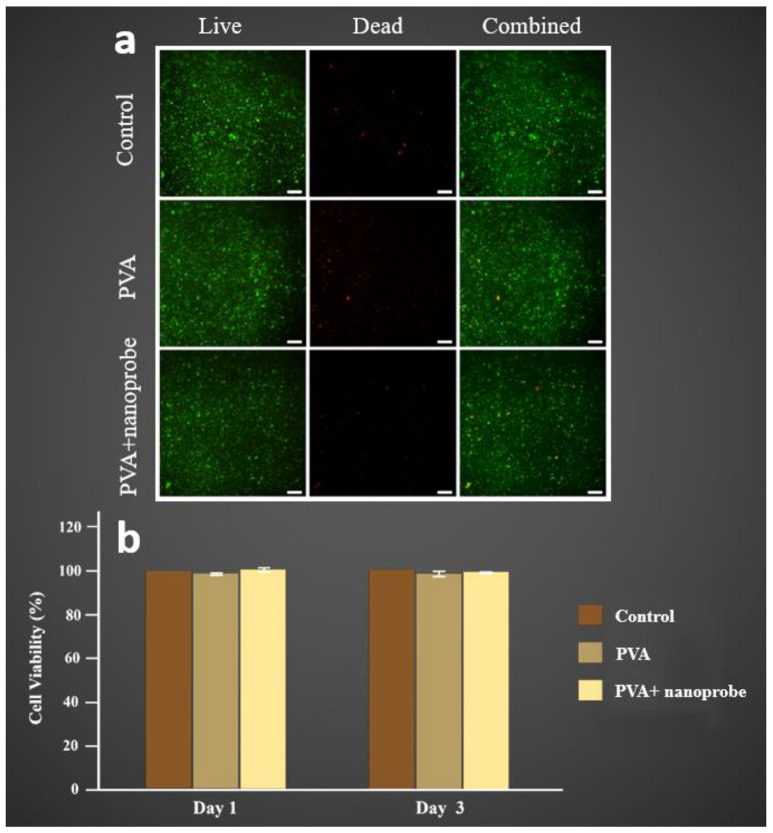
Cell viability: (**a**) live and dead staining of the cells cultured using media with conventional media (Control), PVA, and PVA + nanoprobe (Scale bars = 200 µm). (**b**) Cell viability assay (MTS) in A549 cells after 1 and 3 days. The data represent the means of three independent experiments.

Furthermore, the addition of nanoprobe to the PVA structure did not affect the biocompatibility of the fluorescent microchip. The viability of the cells was checked after 72 h for each group. The cells cultured using the microchips’ dipped media were alive and growing well (Figure 9a). MTS assay was then performed to test the cytotoxicity of the PVA and PVA + nanoprobe microchips on day 1 and day 3. Viable mammalian cells could reduce the MTS tetrazolium compound to coloured formazan dye that was soluble in cell culture by NAD(P)H-dependent dehydrogenase enzymes. The results suggested that both the PVA and PVA + nanoprobe microchips were not toxic to A549 cells. As shown in Figure 9b, compared to the control populations, 98.6% and 98.7% of cells survived when cultured in media dipped with PVA alone, and 100.61% and 99.91% cells survived when cultured in media with PVA + nanoprobe microchip.

The preparation of AIE aggregates mainly involves precipitation processes leading to the production of fluorescent aggregates of small molecules [65]. While AIE aggregates using low molecular weight molecules have been widely used for different applications in materials science and technology at the microscale, the creation of highly efficient AIEgens to enhance the fluorescent property of materials at the macroscale is still challenging [66]. Of particular significance, traditional methods to create fluorescent hydrogels, i.e., soaking, destabilizing the chemical structure, and altering the mechanical property [67]. Studies have shown that the development of high molecular weight AIE systems may resolve the issue [68]; however, the synthesis of AIE polymers is typically difficult, time-consuming involving multi-step processes [69], expensive, and requires different polymerization reactions and catalysts [70]. The current study proposed a novel, cost-effective, and straightforward approach to developing an AIE-HB fluorescent nanoprobe, relying on the formation of hydrogen bonds between the AIE and HB. The challenge to effectively optimise the binding strength to tune the fluorescence property of the AIE-HB nanoprobe was addressed using a VFD and the effect of HB types was evaluated. This strategic approach was successfully employed to create fluorescent injectable bioadhesive gels with the enhanced self-adhesion property as an injectable AIEgen delivery system. Moreover, the nanoprobe that was readily prepared in this study can be used for fabricating fluorescent microchips with superior biomedical and mechanical properties for cell culture studies. Deep penetration of smaller AIE nanoparticles into the hyperbranched structure might occur readily by using a VFD to produce AIE-HB nanoprobes. Consequently, the restriction of the intermolecular motion of the AIE inside the HB structure was more likely to occur by the formation of hydrogen bonds, leading to higher fluorescence properties. The HB generation 4 featured more side branches and a higher number of OH groups per molecule compared to generations 3 and 2, leading to the creation of more hydrogen bonds and the development of a more stable AIE-HB assembly. This was confirmed by the measurement of the equilibrium dissociation constants and the stability of the nanoprobes in this study. Compared to the AIE alone, the introduction of the nanoprobe into the PVA structure provided more hydroxyl groups facilitating the formation of hydrogen bonds between the AIE-HB and PVA molecules. Besides, the nanoprobe with a branched molecular structure and bigger size compared to the AIE alone was more likely to be physically secured within the PVA structure.

## 3. Experimental Section

### 3.1. Materials

PVA (Mowiol^®^ 28-99, CAS No: 9002-89-5, molecular weight 145,000 and degree of hydrolysis >99%), dimethyl sulfoxide (DMSO), hyperbranched polymer, bis-MPA polyester-n-hydroxyl, with n = 16, 32, and 64 representing generations 2, 3, and 4, respectively (CAS no: 326794-48-3) were purchased from Sigma-Aldrich, Sydney, Australia. TPE-2BA was purchased from AIEgen Biotech Co., Ltd., Hong Kong, China. A549 cells were purchased from the American Type Cell Culture Collection (ATCC, Rockville, MD, USA). Dulbecco’s Modified Eagle Medium (DMEM) supplemented with 10% fetal bovine serum (FBS) and 1% L-glutamine were prepared from Gibco. Trypsin/EDTA was purchased from Sigma-Aldrich. Live/dead cell double staining and CellTiter 96^®^ Aqueous One Solution Cell Proliferation Assay kits were purchased from Sigma-Aldrich (Sydney, Australia) and Promega, Madison (Sydney, Australia), respectively. Mouse L929 fibroblast cell line was purchased from the European Collection of Cell Cultures (ECACC).

### 3.2. AIEgen-Hyperbranched Polymer (AIE-HB) Preparation and Characterization

AIE and HB solution: TPE-2BA, a derivative of tetraphenylethylene with 2 boronic acid groups, was used as an AIEgen. AIE solution was prepared by dissolving 0.105 g (250 µmol) of TPE-2BA in 25 mL DMSO to reach a final concentration of 10 mM and served as a stock solution. The stock solution was stored at 4 °C for further use. Appropriate amounts of HBs with different generations were dissolved in 200 mL DMSO to prepare HB solutions (1 mM).

Preparation of TPE-2BA aggregate (AIEgen aggregates): an appropriate amount of stock solution was diluted in DMSO to reach a final concentration of 50 µM. A total of 200 µL of AIE solution (50 µM) was added dropwise to 1800 µL water under constant stirring to prepare AIE aggregates at water fraction = 90%.

Preparation of AIE-HB assembly using the traditional method (mixing): proper amounts of the AIE stock solution were diluted with the HB solutions, reaching the final concentration of AIE in the solution = 50 µM. The concentrations of AIE (50 µM) and HB (1 mM) remained constant. Then, 200 µL of AIE-HB solution was added dropwise to 1800 µL water under constant stirring to prepare AIE-HB aggregates at water fraction = 90%.

Preparation of the AIE-HB nanoprobe using VFD: 2700 mL of water was added to the VFD tube, and the rotation speeds were set from 1000 to 5000 RPM (step 1000 RPM). At each rotation speed, 300 mL of AIE-HB solution (in DMSO) was injected into the VFD rotating tube to prepare VFD-driven AIE-HB nanoprobes at water fraction = 90%.

Isothermal titration calorimetry (ITC): ITC was employed (Nano ITC, TA Instrument, New Castle, DE, USA) to measure the binding constant between AIE and HB with different generations. ITC was performed at 4 °C, with 25 µL of AIE solution (50 µM) was injected into 1 mM HB solutions (generations 2, 3, and 4) in equal steps of 2.5 µL.

Measurement of fluorescent property and stability of the AIE-HB nanoprobe: both the fluorescent spectrum and the fluorescence stability for AIE-HB nanoprobes at WF = 90% were identified using a spectrophotometer (Agilent Scientific Instruments, Melbourne, Australia) at excitation and emission wavelengths of 310 and 465 nm, respectively. The fluorescence intensity at the excitation wavelength of 310 nm was recorded for as-prepared samples and at different time points to measure the stability of fluorescence.

### 3.3. Fluorescent Gel Preparation and Characterization

Preparation of injectable bioadhesive gel: PVA solution (6% w/w) was prepared by dissolving PVA powder in distilled water at 80 °C under constant stirring for 3 h. An AIE-HB nanoprobe, with optimum fluorescent properties, was mixed with PVA solution at the ratio of 20% (w/w) using a VFD. For this, the PVA solution was placed in the VFD tube, and the rotation speed was set to 4000 RPM. Gradually, the AIE-HB nanoprobe was added to the tube, and micromixing under shear was performed for 5 min. To develop injectable fluorescent PVA gel, a clean VFD tube was filled with 2 mL of the borax solution in water (50 mM), VFD rotation speed was set to 4000 RPM, and PVA + nanoprobe was injected into the VFD via the inlet feed jet using a 5 mL syringe (Figure 1a). The formation of the injectable gel was continued for 5 min before extraction. Further, the concentration of borax was increased two-fold (100 mM) and PVA fluorescent gels were prepared using the VFD (as described above) and traditional mixing methods. For traditional mixing, a magnet stirrer (1000 RPM at room temperature) was used to mix borax with PVA + AIE nanoprobe. Both VFD-driven and traditionally mixed samples were then dried at room temperature for 1 h to perform self-adhesion tests.

Self-adhesion measurement of bioadhesive gels: the self-adhesion property was measured for the injectable fluorescent PVA gel. A micromechanical machine (CellScale, Canada) was employed to compare the self-adhesion strength of different samples, as previously described [58]. Briefly, gels with a 2 mm width and 10 mm length were prepared and cut in half. Each half was placed in the CellScale actuators, and samples were moved carefully towards each other until the compression force at the cutting edge of the samples was 30 mN. Then, a displacement control tensile test was executed at the strain rate of 5% s^−1^ for 460 s to measure the self-healing property.

Nanoprobe release from injectable bioadhesive gel: the release of nanoprobe from the injectable bioadhesive PVA gel was quantified by the measurement of the fluorescent properties of media over time using a UV spectrophotometer (Agilent Scientific Instruments, Australia) at an excitation wavelength of 310 nm. The fluorescent gel (5 mL) was prepared as explained above and was dried at 37 °C using an oven. The dried gel was then placed in a beaker (100 mL) containing water + DMSO (90% water fraction) solution. Samples from the release media were collected at different time points until the fluorescent maxim remained unchanged. The collected release media was then returned to the beaker.

### 3.4. Fabrication and Characterization of Fluorescent Microchip

Microchip fabrication using 3D printing: a complex 3D platform containing different patterns was prepared using 3D printing technology. SolidWorks software was used to prepare the design and the final STL file was exported to the 3D printer software (MiiCraft 125, Version 4.01, MiiCraft, Inc, Jena, Germany). A digital light processing (DLP) 3D printer (MiiCraft Ultra 50, MiiCraft) with XY resolution of 10 μm, was used to fabricate the platform with a printing area of 100 mm^2^. The print options were carefully adjusted allowing 3D printing of features with high resolution and a base layer was used to ensure the platform adhesion to the picker for the duration of the print. The DLP process was carried out under 385–405 nm UV wavelength and the curing time for the resin (BV-007) was set to 24 s. A buffer layer was utilised to facilitate the transition from the base layer into the printed platform. The 3D printed platform was then used as a mold to fabricate the microchip with specific patterns using the film casting method. PVA + AIE nanoprobe and PVA solution (control) were prepared as described above. The 3D printed platform containing hydrogels was then placed in an oven at 60 °C for 24 h to dry and then were peeled off the platform.

Microchip mechanical properties: the failure mechanical properties of microchips made of PVA and PVA + nanoprobe (as prepared) were measured using an ElectroForce BioDynamic test instrument (5110, TA Instrument). The average thickness of the samples (W × L = 10 mm × 40 mm) was 1.24 ± 0.31 mm measured at five different points using a digital caliper. To execute mechanical characterization, samples were secured at both ends of grippers with their initial length set at 2 mm. The mechanical tests were conducted at room temperature up to failure with a strain rate of 0.1 mm.min^−1^. Stress–strain data were used to calculate young’s modulus, elongation at break, and toughness.

Swelling characteristics of microchips: the swelling ratio was measured to characterise the swelling behaviour of PVA and PVA + nanoprobe microchips. Dry samples were immersed in distilled water and the amount of water uptake was measured gravimetrically at different time points. Samples were weighed every five minutes for the initial 45 min, after which the time was extended to 30 min and 1 h. Before each measurement, samples were gently wiped with tissue paper to remove excess surface water. The swelling ratio was calculated using Equation (1):

(1) $$Swelling\;ratio\;\left(SR\right)\%=\frac{M_{t}-M_{0}}{M_{0}}\times 100$$

where *M_t_* and *M*_0_ were the weight of the wet and dried samples, respectively.

Stability of microchips in water and cell culture media: to examine the stability, both PVA and PVA + nanoprobe microchips were swelled (in water) to their maximum swelling ratio in a petri dish, and their structural stability was evaluated using a light microscope (Olympus IX73,Tokyo, Japan). To evaluate the stability of microchips in cell culture media, a mouse L929 fibroblast cell line was grown in DMEM containing 10% (*v/v*) FBS (Gibco, thermos Fisher, Waltham, MA, USA), 1% (*v/v*) penicillin–streptomycin (10,000 units·mL^−1^–10,000 μg·mL^−1^), and 2 mM L-glutamine. When cells were approximately 80% confluent, the media was removed, and the cells were then washed with 10 mL Dulbecco’s Phosphate Buffered Saline (DPBS) (without calcium and magnesium) to remove any remaining media. Thereafter the DPBS was replaced with 2 mL of a 0.25% (*v/v*) trypsin/EDTA, and the cells were incubated at 37 °C for 5 min at room temperature. The cell suspension was transferred into a sterile universal tube containing 10 mL cell culture media containing 10% (*v/v*) FBS to neutralise the trypsin and centrifuged at 150× *g* for 5 min. The waste media containing trypsin was removed, and the pellet of cells was re-suspended in the appropriate cell culture media to obtain L929 fibroblasts cells with a concentration of 0.5 × 10^5^ cells mL^−1^. Subsequently, L929 fibroblasts cells were loaded on the PVA + nanoprobe microchip and were incubated in a 5% (*v/v*) CO_2_ atmosphere at 37 °C to allow the microchip’s stability evaluation using a light microscope (Olympus IX73, Tokyo, Japan).

Nanoprobe leakage from microchip: to quantify the leakage of nanoprobe from the PVA + nanoprobe microchip, it was placed in a beaker (100 mL water) to swell and samples from the swelling media were collected at different time points. The fluorescent spectra of the dry microchip and swelling media were collected using a spectrophotometer at emission wavelengths of 310 nm (Agilent Scientific Instruments, Melbourne, Australia) to measure the leakage of the nanoprobe. The collected swelling media was then returned to the beaker.

Imaging: PVA solutions including AIE and nanoprobe were dropped cast on the microscope slides and fluorescent images were captured using an AX70 upright fluorescent microscope (Olympus, Japan with Zen Blue Image capture software). For SEM imaging (Inspect F50, FEI, Hillsboro, Oregon USA), dropped cast films were dried in a vacuum oven at 37 °C and coated with platinum using a dual-target sputter coater (Q300T-D, Quorum, UK). SEM images were captured under low voltage (kV = 5). Images to qualitatively identify the fluorescent properties of microchips were captured using a fluorescent microscope (DAPI channel—Olympus IX73, Tokyo, Japan). Open-source FIJI (ImageJ) was used to identify the impact of the microchips’ surface morphology on the orientation and shape of the cells. OrientationJ plugin was used to measure the orientation of cells in the input binary (8-bit) microscope images. Cell orientation analysis was conducted with Cubic Spline Gradient (sigma = 5) selected as the structural tensor for fitting the data.

Microchip cell viability and proliferation: to test the biocompatibility, all the PVA and PVA + nanoprobe microchips were exposed to UV light for 30 min before dipping in 6 well plates filled with culture media and incubated for 48 h. A lung cancer cell line (A549) was cultured in DMEM supplemented with 10% FBS and 1% L-glutamine in a 6 well plate with a seeding density of 300,000 cells per well. In the first plate, the culture media dipped with the PVA sample was used. The media dipped with PVA + nanoprobe sample was used in the second plate, whereas the conventional media was used as the control in the third plate. Microscope Live/dead cell double staining kit (Sigma-Aldrich, Australia) was used to check the viability of the cells. The assay solution of the stain was prepared by adding 10 μL of Calcein-AM (Solution A) and 5 μL of propidium iodide (Solution B) in 5 mL of PBS. After washing with PBS, the assay solution was incubated at 37 °C for 15 min. Olympus IX73 Inverted Microscope was used to simultaneously observe live and dead cells.

The cytotoxicity of the PVA and PVA + nanoprobe microchips were assessed using the CellTiter 96^®^ Aqueous One Solution Cell Proliferation Assay kit (Promega, Madison, WI, USA) according to the manufacturer’s instructions for MTS assay. The A549 cells were seeded on 96-well culture plates. After 24 h, the media was removed, and the cells were washed with PBS. A total of 20 µL of MTS reagent was added to each culture well and incubated in the dark for 3 h at 37 °C. The assay was also performed after 72 h of cell culture. Empty wells with media only alone were used as blanks to subtract absorption by media components. The absorption was measured (at a wavelength of 490 nm) using a fluorescence plate reader (Infinite 200 PRO; TECAN). The data were expressed as the percentage of cell viability compared with the control.

## 4. Conclusions

The current study, utilizing a VFD, presented an innovative method for the fabrication of AIE-HB nanoprobes. While different generations (2, 3, and 4) of HBP were mixed with TPE-2BA (AIE) to prepare AIE-HB nanoprobes, our results revealed that the use of HB generation 4 creating a brighter nanoprobe with enhanced maximum fluorescent properties. In contrast to traditional methods involving polymerization and post-functionalization to develop fluorescent hyperbranched molecules from scratch, our proposed method offers a one-step method to prepare an AIE-HB nanoprobe. Our further investigations revealed the efficiency of the AIE-HBP nanoprobe in developing fluorescent injectable gel and a microchip for cell culture. We observed that the introduction of the nanoprobe to the PVA structure induced more opportunities to form hydrogen bonds, leading to the development of a fluorescent PVA bioadhesive gel that effectively controlled the release of the AIE. This finding suggested that the injectable bioadhesive gel can potentially be used as a carrier for AIEgen delivery. Moreover, it was found that the addition of the AIE nanoprobe to the PVA hydrogel (physical crosslinking) to fabricate a microchip for cell culture, tuned the mechanical properties and increased the dimensional stability. Furthermore, it was observed that the addition of nanoprobe to the PVA structure did not affect the biocompatibility of the fluorescent microchip. The final nanoprobe with a high number of free hydroxyl groups, compared to the AIE, has a great potential to be used for the fabrication of fluorescent injectable bioadhesive gels and microchips for biomedical applications with hydrogen bonding, effectively shaping their final structural stability and properties.

## Data Availability

The data presented in this study are available on request from the corresponding authors.
